# The experience of high-flow nasal cannula in hospitalized patients with 2019 novel coronavirus-infected pneumonia in two hospitals of Chongqing, China

**DOI:** 10.1186/s13613-020-00653-z

**Published:** 2020-03-30

**Authors:** Ke Wang, Wei Zhao, Ji Li, Weiwei Shu, Jun Duan

**Affiliations:** 1 Department of Respiratory and Critical Care Medicine, The Second Affiliated Hospital of Chongqing Medical University, Chongqing, 400010 People’s Republic of China; 2 Department of Oncology, The Second Affiliated Hospital of Chongqing Medical University, Chongqing, 400010 People’s Republic of China; 3 Department of Thoracic Surgery, Chongqing Public Health Medical Center, Chongqing, 500106 People’s Republic of China; 4 Department of Critical Care Medicine, Yongchuan Hospital of Chongqing Medical University, Xuanhua Road 429, Yongchuan District, Chongqing, 402160 People’s Republic of China; 5 Department of Respiratory and Critical Care Medicine, The First Affiliated Hospital of Chongqing Medical University, Youyi Road 1, Yuzhong District, Chongqing, 400016 People’s Republic of China

**Keywords:** Coronavirus, Pneumonia, High-flow nasal cannula

## Abstract

**Background:**

The outbreak of a novel coronavirus (2019-nCoV)-infected pneumonia (NCIP) is currently ongoing in China. Most of the critically ill patients received high-flow nasal cannula (HFNC) oxygen therapy. However, the experience of HFNC in this population is lacking.

**Methods:**

We retrospectively screened 318 confirmed patients with NCIP in two hospitals of Chongqing, China, from January 1st to March 4th, 2020. Among them, 27 (8.4%) patients experienced severe acute respiratory failure including 17 patients (63%) treated with HFNC as first-line therapy, 9 patients (33%) treated with noninvasive ventilation (NIV) and one patient (4%) treated with invasive ventilation. HFNC failure was defined by the need of NIV or intubation as rescue therapy.

**Results:**

Of the 17 HFNC patients, 7 (41%) experienced HFNC failure. The HFNC failure rate was 0% (0/6) in patients with PaO_2_/FiO_2_ > 200 mm Hg vs. 63% (7/11) in those with PaO_2_/FiO_2_ ≤ 200 mm Hg (*p* = 0.04). Compared with baseline data, the respiratory rate significantly decreased after 1–2 h of HFNC in successful group [median 26 (IQR: 25–29) vs. 23 (22–25), *p* = 0.03]. However, it did not in the unsuccessful group. After initiation of NIV as rescue therapy among the 7 patients with HFNC failure, PaO_2_/FiO_2_ significantly improved after 1–2 h of NIV [median 172 (150–208) mmHg vs. 114 (IQR: 79–130) under HFNC, *p* = 0.04]. However, two out of seven (29%) patients with NIV as rescue therapy ultimately received intubation. Among the 27 patients with severe acute respiratory failure, four patients were eventually intubated (15%).

**Conclusions:**

Our study indicated that HFNC was the most common ventilation support for patients with NCIP. Patients with lower PaO_2_/FiO_2_ were more likely to experience HFNC failure.

## Introduction

In December 2019, acute respiratory infection due to 2019 novel coronavirus (2019-nCoV), now known as novel coronavirus-infected pneumonia (NCIP), emerged in Wuhan, China [1, 2]. The main symptoms were fever, cough, dyspnea, myalgia, fatigue, and radiographic evidence of pneumonia [2–4]. Human-to-human transmission of NCIP has been reported, even in the incubation period [5–7]. In a hospital, 29% of health care workers and 12% of patients who were already hospitalized for other reasons have been identified as presumed hospital-related transmission and infection [4]. The NCIP has spread worldwide and many countries have reported cases of NCIP [8–11]. As of February 11, 2020, 44,672 cases with NCIP were confirmed and 1023 cases died in China [12]. The WHO has declared the outbreak of NCIP as a Public Health Emergency of International Concern on January 30, 2020.

In the hospitalized NCIP patients, the time from disease onset to shortness of breath was median 8 days and to development of ARDS was median 10.5 days [2]. And the rate of development of ARDS ranged from 20 to 29% [2, 4]. Most of the patients received oxygen therapy. High-flow nasal cannula (HFNC) is one of the oxygen therapies for critically ill patients [13]. However, to the best of our knowledge, there were no studies to report the use of HFNC in hospitalized NCIP patients. Here, we aimed to report the experience of HFNC in this population.

## Methods

This was a retrospective observational study performed in two hospitals of Chongqing, China. The 2019-nCoV was confirmed by real-time reverse transcription polymerase chain reaction (RT-PCR) assay [4]. The diagnosis of NCIP was based on clinical characteristics, chest imaging and RT-PCR assay. We screened all the patients with NCIP in two hospitals (Yongchuan Hospital of Chongqing Medical University and Chongqing Public Health Medical Center) from January 1st to March 4th, 2020. The NCIP patients who required HFNC, NIV or invasive ventilation to improve oxygen were classified as severe acute respiratory failure. The study protocol was approved by the local ethics committee and institutional review board (approval number 20200201). As this was a retrospective study, the informed consent was waived.

The critically ill patients who received HFNC (Fisher & Paykel, Auckland, New Zealand, or HUMID-BM, Respircae Medical, Shen Yang, China) were managed by their attending physicians. The temperature was set at 31 to 37 °C, the flow was set at 30 to 60 L/min, and the fraction of inspired oxygen concentration (FiO_2_) was set to maintain the SpO_2_ more than 93%. The continuous use of HFNC was required for all the patients at the initial phase. When the respiratory failure was reversed, the intermittent use of HFNC was performed. We gradually increased the time of standard oxygen and shortened the duration of HFNC until the HFNC was totally weaned. However, if the respiratory failure progressively deteriorated, the attending physicians determined to use noninvasive ventilation or invasive mechanical ventilation as a rescue therapy. HFNC failure was defined by the need of NIV or intubation as rescue therapy.

Before the use of HFNC, we collected the demographics, vital signs, laboratory tests and the arterial blood gas tests. The baseline PaO_2_/FiO_2_ was measured under standard oxygen just before HFNC. The FiO_2_ was estimated as follows: FiO_2_ (%) = 21 + 4 * flow (L/min) [14]. We also assessed the disease severity by acute physiology and chronic health evaluation II (APACHE II) score and organ failure by sequential organ failure assessment (SOFA) score. At 1–2 h and termination of HFNC, we also collected the vital signs and arterial blood gas tests. Among the patients who experienced HFNC failure and needed NIV as rescue therapy, these variables were also collected at 1–2 h and termination of NIV.

Continuous variables were reported as mean value (standard deviation) or median value [interquartile range (IQR)] when appropriate. The differences between two groups were analyzed by Student’s *t* test or Mann–Whitney *U* test. The differences between different time points within group were analyzed by the use of paired Student’s *t* test. Categorical variables were reported as number and percentage, and analyzed using the Chi-squared test or Fisher’s exact test. A *p* value < 0.05 was considered significant.

## Results

**Fig. 1:**
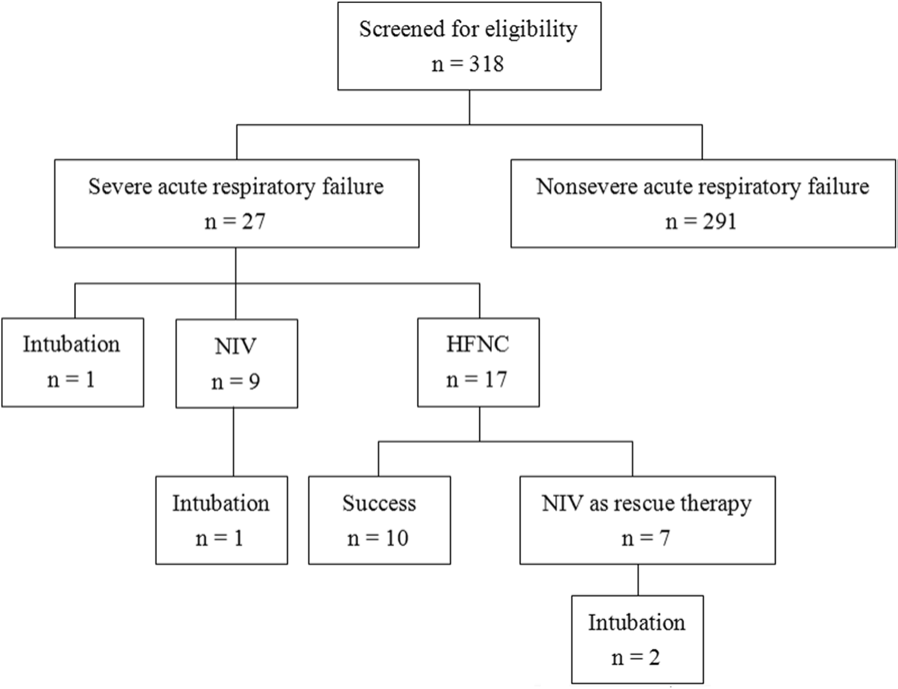
Flow of patient screening and enrollment

We screened 318 patients with NCIP for eligibility (Fig. 1). Twenty-seven out of 318 (8.4%) patients experienced severe acute respiratory failure. Among the patients with severe acute respiratory failure, HFNC was used as first-line therapy in 17 (63%) patients, noninvasive ventilation (NIV) in 9 (33%) patients, and invasive ventilation in one (4%) patient. Four patients were eventually intubated (15%). The characteristics of the 17 patients treated with HFNC as first-line therapy are summarized in Table 1.

**Table 1:** Clinical characteristics of the enrolled patients

	Total cohort *N* = 17
Age, years	65 (56–75)
Male (%)	7 (41%)
APACHE II score	8 (5–11)
SOFA score	3.0 (2.5–3.5)
Duration of HFNC, hours	76 (34–186)
Comorbidity	
Hypertension	3 (18%)
Diabetes mellitus	3 (18%)
Chronic heart disease	3 (18%)
Laboratory tests	
White blood cell counts, × 10^9^/L	5.4 (4.2–7.1)
Lymphocyte count, × 10^9^/L	0.7 (0.5–0.9)
Platelet counts, × 10^9^/L	154 (121–259)
Hemoglobin, mg/dL	128 (118–138)
Albumin, g/L	35 (32–38)
Potassium, mmol/L	3.9 (3.5–4.2)
Sodium, mmol/L	136 (135–138)
Chlorine, mmol/L	101 (99–105)
Creatinine, μmol/L	60 (53–69)
Total bilirubin, μmol/L	11 (10–15)
C-reactive protein, mg/L	39 (22–67)
Procalcitonin, ng/mL	0.07 (0.06–0.09)

*APACHE II* acute physiology and chronic health evaluation II, *SOFA* sequential organ failure assessment

**Fig. 2:**
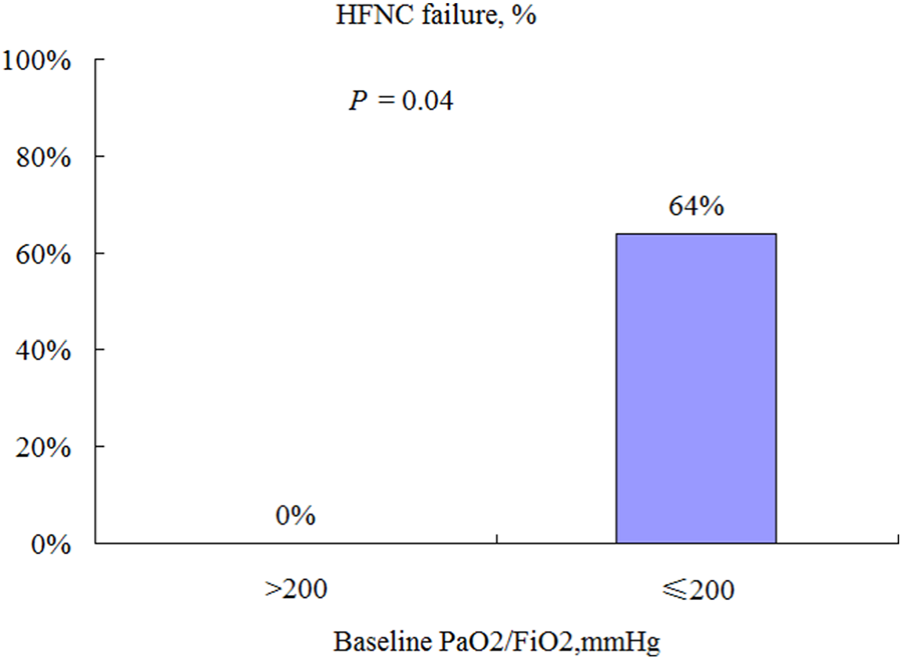
HFNC failure rate in different groups stratified by PaO_2_/FiO_2_

Among the 17 patients treated with HFNC, 7 (41%) experienced HFNC failure and needed NIV as a rescue therapy. Two out of seven (29%) patients were subsequently intubated after NIV failure. At baseline, the number of patients with PaO_2_/FiO_2_ > 200 and ≤ 200 mmHg was 6 and 11, respectively. No HFNC failure occurred in patients with PaO_2_/FiO_2_ > 200 mmHg (Fig. 2). However, the failure rate was 64% in patients with PaO_2_/FiO_2_ ≤ 200 mmHg.

**Fig. 3:**
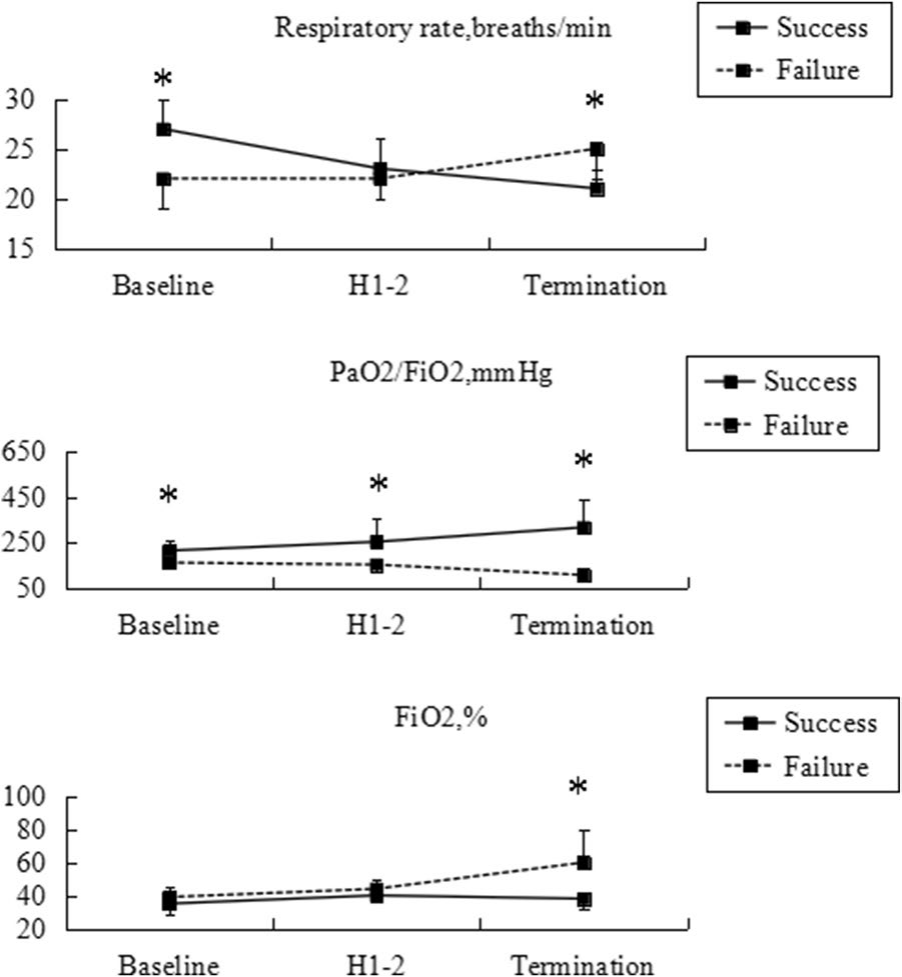
Respiratory rate, PaO_2_/FiO_2_ and FiO_2_ at baseline, 1–2 h and termination of HFNC. *p < 0.05 for comparisons between two groups

The comparisons between patients with HFNC success and failure are summarized in Table 2 and Fig. 3. Compared with baseline data, the respiratory rate significantly decreased after 1–2 h of HFNC in successful group [median 26 (IQR: 25–29) vs. 23 (22–25), *p* = 0.03]. However, it did not in the unsuccessful group. After initiation of NIV as rescue therapy, the PaO_2_/FiO_2_ improved after 1–2 h of NIV [median 114 (IQR: 79–130) vs. 172 (150–208) mmHg, *p* = 0.04] (Table 3).

**Table 2:** Vital signs and arterial blood gas tests at baseline and 1–2 h of HFNC

	HFNC success *N* = 10	HFNC failure *N* = 7	*p*
Baseline			
RR, breaths/min	26 (25–29)	23 (21–23)	0.02
HR, beats/min	84 (73–97)	79 (74–85)	0.40
SBP, mmHg	125 (121–138)	108 (105–133)	0.07
DBP, mmHg	73 (71–78)	63 (60–76)	0.07
pH	7.43 (7.39–7.47)	7.46 (7.44–7.48)	0.05
PaCO_2_, mmHg	38 (35–40)	34 (32–36)	0.13
PaO_2_/FiO_2_, mmHg	223 (161–252)	159 (137–188)	0.02
FiO_2_, %	34 (32–41)	41 (33–41)	0.24
1–2 h of HFNC			
RR, breaths/min	23 (22–25)	23 (21–23)	0.36
HR, beats/min	82 (71–92)	85 (78–92)	0.91
SBP, mmHg	118 (111–131)	110 (106–139)	0.73
DBP, mmHg	71 (70–78)	65 (63–78)	0.36
pH	7.46 (7.42–7.49)	7.47 (7.44–7.47)	0.41
PaCO_2_, mmHg	38 (36–39)	34 (33–35)	0.06
PaO_2_/FiO_2_, mmHg	209 (179–376)	142 (130–188)	0.03
FiO_2_, %	40 (35–40)	40 (40–50)	0.06

*RR* respiratory rate, *HR* heart rate, *SBP* systolic blood pressure, *DBP* diastolic blood pressure, *HFNC* high-flow nasal cannula

**Table 3:** Vital signs and arterial blood gas tests at termination of HFNC and 1–2 h of NIV as rescue therapy among the patients with HFNC failure

	Termination of HFNC	1–2 h of NIV as rescue therapy	*p*
RR, breaths/min	23 (22–29)	24 (21–24)	0.12
HR, beats/min	74 (67–89)	79 (74–88)	0.96
SBP, mmHg	133 (111–138)	125 (111–130)	0.34
DBP, mmHg	72 (68–76)	71 (68–82)	0.93
pH	7.49 (7.46–7.50)	7.49 (7.48–7.50)	0.20
PaCO_2_, mmHg	33 (32–36)	32 (30–34)	0.04
PaO_2_/FiO_2_, mmHg	114 (79–130)	172 (150–208)	0.04
FiO_2_, %	50 (40–70)	50 (40–70)	0.10

*RR* respiratory rate, *HR* heart rate, *SBP* systolic blood pressure, *DBP* diastolic blood pressure, *HFNC* high-flow nasal cannula, *NIV* noninvasive ventilation

## Discussion

To the best of our knowledge, there were no studies to report the use of HFNC in patients with NCIP. Our study originally reported that HFNC was the most common ventilation strategies for NCIP patients. Patients with lower PaO_2_/FiO_2_ were more likely to experience HFNC failure. Forty-one percent of patients required NIV as rescue therapy. However, 29% of NIV patients ultimately received intubation.

In our study, we found that the number of HFNC patients were much higher than NIV patients when the HFNC or NIV was used as an initial oxygen support. It means that physicians were more likely to use HFNC among the critically ill patients caused by NCIP. As the outbreak of NCIP in China, thousands of clinical staff joined in the patient management. Most of them had no experience on how to use HFNC or NIV. The current knowledge shows that (1) the HFNC is non-inferior to NIV on intubation rate in critically ill patients [15]; (2) the use of HFNC is more comfortable than NIV and the skin breakdown is less likely to occur [16, 17]; and (3) the manipulation of HFNC is much easier than NIV. Therefore, the clinical staff were more likely to use HFNC in NCIP patients.

Person-to-person transmission of NCIP has been confirmed. In the early stages, the epidemic doubled in size every 7.4 days, and the estimated basic reproductive number was 2.2 (95% CI 1.4 to 3.9) [5]. The virus is believed transmitted mostly via droplets or contact and possibly via aerosol [18]. All people are generally susceptible to the virus. As of February 11, 2020, 1716 clinical staff have been infected with NCIP, and 5 of them died [12]. Therefore, a device that produces lesser number of droplets or aerosol is required. The exhaled air dispersion produced by HFNC was limited and the risk of hospital-acquired infection did not increase [19, 20]. Therefore, the use of HFNC in NCIP patients is feasible. However, the amount of condensation in the circuit increased when the ambient temperature decreased [21]. The condensed water became an important source of infection for NCIP. So, avoidance or reduction of condensation was very important when the HFNC was used.

A previous study reported that 38% of HFNC patients required intubation [13]. In this study, 13% of patients experienced HFNC failure and required NIV as rescue therapy. Among the NIV patients who experienced HFNC failure, the intubation rate was 64%. However, in our study, 41% of patients experienced HFNC failure. Among the unsuccessful patients, all of them directly switched to NIV (no one directly switched to intubation). It means that the physicians who managed the NCIP patients were more likely to use NIV than intubation when the HFNC was unable to maintain the oxygenation. We speculated that the process of intubation made the physicians at high risk of infection because of the close encounter and irritable cough. However, among the patients with HFNC failure in our study, only 29% received intubation. This indicates that the success rate is high after transition to NIV.

Our study has several limitations. This is a retrospective observational study. We did not predefine how to manage the HFNC. The transition to NIV or intubation was decided by the attending physicians. Different physicians have different opinions on the point to switch to NIV or intubation. However, this study can reflect on how the HFNC has been used in the real world among the NCIP patients. In addition, we only enrolled 17 patients in this study as the enrollment period is short. To our knowledge, there are no studies that report on how the HFNC was used in NCIP patients. Rapid publication is very important for public health. It also can provide an important reference for clinical physicians when using HFNC in NCIP patients.

## Conclusions

This study firstly reports the experience of how to use HFNC in patients with NCIP. HFNC was the most common ventilation support for patients with NCIP. Patients with lower PaO_2_/FiO_2_ were more likely to experience HFNC failure. The overall rate of intubation was 15% among the NCIP patients with severe acute respiratory failure.

## Data Availability

The datasets analyzed during the current study are available from the corresponding author on reasonable request.

## Abbreviations

NCIP: Novel coronavirus (2019-nCoV)-infected pneumonia
HFNC: High-flow nasal cannula
NIV: Noninvasive ventilation
ARDS: Acute respiratory distress syndrome
APACHE II: Acute physiology and chronic health evaluation II
SOFA: Sequential organ failure assessment
RR: Respiratory rate
HR: Heart rate
SBP: Systolic blood pressure
DBP: Diastolic blood pressure
IQR: Interquartile range
